# Exercise training preferentially modulates α1D-adrenergic receptor expression in peripheral arteries of hypertensive rats

**DOI:** 10.1515/biol-2025-1232

**Published:** 2025-12-30

**Authors:** Ling-Zhi Li, Meng Li, Tong Deng, Yue Ma, Lu Li, Jia-Li Qi, Yu-Qi Jiang

**Affiliations:** Pain Department, Affiliated Hospital of Hebei University, Baoding, 071000, China; Department of Anesthesiology, Affiliated Hospital of Hebei University, Baoding, 071000, China

**Keywords:** blood vessels, exercise, receptors, SHR rats, WKY rats

## Abstract

To evaluate the relative expression of α1D, P2X1, and P2Y6 receptor mRNAs in arterial tissues (renal artery [RA], thoracic aorta [Aor], and caudal artery [Cau]) of spontaneously hypertensive rats (SHR) and Wistar Kyoto rats (WKY), and to assess exercise-induced changes, SHR and WKY rats were assigned to sedentary (S) or exercise training (E) groups. The E group underwent 16 weeks of treadmill training. Systolic blood pressure (SBP) decreased significantly in SHR-E rats. Receptor mRNA expression followed P2X1 > α1D > P2Y6, with P2X1 highest in the Cau and α1D highest in the Aor. SHR rats showed lower expression in all arteries compared to WKY. Exercise training had a greater effect on α1D receptors, while P2X1 remained predominant. In SHR-E rats, expression decreased for all receptors except α1D in the Cau, which increased. The distribution of α1D and P2X1 receptors correlates with vascular diameter and hypertension. Exercise impacts α1D receptors more, particularly in peripheral arteries. P2X1 dominance in peripheral arteries persists post-exercise, possibly aiding blood redistribution during exercise and maintaining organ function.

## 1 Introduction

Hypertension is a global public health issue that can lead to severe cardiovascular and cerebrovascular complications. Elevated arterial blood pressure is closely associated with increased vascular resistance due to the contraction of vascular smooth muscle cells (VSMC). Abnormalities in sympathetic nerve activity (SNA) and myogenic tone (MT), as well as changes in the structure of the vascular wall, are important causes of primary hypertension [1]. Exercise training is an important non-pharmacological intervention for the prevention and treatment of hypertension, and the latest hypertension guidelines emphasize the role of personalized exercise training in the management of hypertension [2].

Within the sympathetic nervous system, cardiovascular function is regulated by endogenous catecholamines through the adrenergic receptor family. Among these, α1-adrenergic receptors (α1-ARs) play a dominant role in regulating vascular smooth muscle contraction. Blood pressure regulation through sympathetic nerves is primarily facilitated through α1D-adrenergic receptor (α1D-AR) and α1A-adrenergic receptor (α1A-AR)-mediated peripheral vasoconstriction. The α1B-adrenergic receptor is located in cardiomyocytes and has a relatively minor role in influencing vascular tone, primarily participating in the regulation of the other two receptor subtypes. While α1D-ARs are highly sensitive to catecholamines. Activation of this receptor subtype enables the maintenance of vascular contractile tension even after the removal of agonists, preventing vascular tone from fluctuating in response to changes in circulating plasma catecholamine levels. This mechanism contributes to circulatory stability and the preservation of organ perfusion.

Within the purinergic system, Adenosine Triphosphate (ATP) is released as a co-transmitter from sympathetic nerve terminals and activates P2X receptors (primarily P2X1 receptors) expressed on vascular smooth muscle. Extracellular nucleotides participate in local blood flow regulation through P2X1 receptors or pyrimidine-sensitive P2Y receptors, with P2X1 receptors being the primary mediators of vasoconstriction in vascular smooth muscle and thus influencing blood pressure. P2X receptor-mediated smooth muscle depolarization leads to the activation of L-type voltage-dependent Ca^2+^ channels, resulting in vasoconstriction [3]. In sedentary rats with selective α1-adrenergic receptor blockade, the P2X1 receptor becomes the predominant receptor mediating the contractile response to sympathetic stimulation. The P2Y6 receptor, as the primary P2Y receptor mediating vasoconstriction through Uridine Triphosphate (UTP) or Uridine Diphosphate (UDP), lacks the rapid desensitization characteristic of P2X1 receptors [4]. P2Y6 receptors have been shown to mediate vasoconstriction in rat cerebral parenchymal arterioles, mouse mesenteric resistance arteries, and human subcutaneous arteries. Additionally, P2Y6 receptors promote the reuptake of norepinephrine (NA) in the central nervous system, thereby regulating its release. The expression of P2Y6 receptors is positively correlated with age and closely associated with Angiotensin-II-induced hypertension, suggesting a critical role in cardiovascular events [5].

Exercise training does not eliminate the vasoconstrictive effects mediated by α1 receptors in muscle sympathetic nerve activity (MSNA), but it enhances the sympatholytic vasodilatory effects mediated by these receptors. However, with increasing age, the sympatholytic vasodilatory effects gradually weaken, leading to relatively enhanced vasoconstrictive responses during exercise, which in turn results in impaired blood flow distribution and metabolic dysfunction [6]. Exercise training can enhance the activity of phosphodiesterase, promote the hydrolysis of ATP, inhibit the activation of P2 receptors, and increase the expression of P2X1 receptors in skeletal muscle of healthy individuals [7].

In previous studies, long-term exercise was found to alter the expression of P2X1, P2Y1, and P2Y2 receptor messenger RNAs (mRNAs) in various vascular tissues of Wistar Kyoto (WKY) rats and Spontaneously Hypertensive rats (SHR), suggesting that exercise training has a more significant impact on P2Y receptor mRNA expression in both rat strains, potentially lowering blood pressure in SHR by increasing purinergic vasodilatory effects [8]. Building upon these findings, the distribution and alterations of purinergic receptors (P2X1, P2Y6) and adrenergic receptors (α1D) that regulate vasoconstriction in the thoracic aorta (Aor), renal artery (RA), and caudal artery (Cau) of WKY rats and SHR rats before and after long-term exercise were further examined in this study. The aim of these observations is to further characterize receptor-mediated vasoconstriction and vasodilation following long-term exercise, providing a basis for future research on the mechanisms by which low-to moderate-intensity exercise enhances cardiovascular function.

## 2 Materials and methods

### 2.1 Experimental animals

Eight-week-old WKY and SHR rats (12 each), weighing 170–200 g, were obtained from Beijing Vital River Laboratory Animal Technology Co., Ltd. Six WKY and SHR rats were randomly assigned to a sedentary control group (Sedentary, S group) and an exercise training group (Exercise training, E group) each. Rats in the exercise group were randomly numbered and underwent an adaptive treadmill training for 5 days, with sessions lasting 10 min per day at a treadmill speed of 5 m/min. Subsequently, they were engaged in low-to moderate-intensity treadmill training at relatively fixed times, following a regimen of five days per week (Monday to Friday), 60 min per day, at a treadmill speed of 18–20 m/min, for a total duration of 16 weeks. Rats in the sedentary group rats were allowed unrestricted movement but were not engaged in any exercise training. Both groups had *ad libitum* access to food and water.

**Ethical approval**: The research related to animal use has been complied with all the relevant national regulations and institutional policies for the care and use of animals, and has been approved by the Ethics Committee of Hebei University (March 8, 2023).

### 2.2 Experimental methods and detection indicators

#### 2.2.1 Determination of rat body weight and arterial blood pressure

A non-invasive blood pressure system was utilized to measure rat systolic blood pressure (SBP) using the tail-cuff occlusion method. SBP was recorded in millimeters of mercury (mmHg). The body weight of each rat was measured and documented both before grouping and at the end of the exercise training period.

#### 2.2.2 Fluorescence quantitative real-time PCR detection of P2X1, P2Y6, and α1D receptor mRNA expression in different vascular tissues

Preparation of Isolated Arterial Specimens: Rats were anesthetized via intraperitoneal injection of 25 % urethane at a dosage of 1.5 g/kg, and exsanguination was performed through the femoral artery. Aor, RA, and Cau specimens were rapidly excised, stripped of surrounding connective tissue, immediately frozen in liquid nitrogen, and stored at −80 °C for subsequent analysis.

RNA Extraction and Quantitative PCR Analysis: Total RNA was extracted from arterial tissues using TRIzol reagent. Complementary DNA (cDNA) synthesis was performed according to the manufacturer’s protocol using the HiScript III RT SuperMix for quantitative (qPCR) kit, and fluorescence quantitative real-time PCR (RT-qPCR) analysis was performed using the Taq Pro Universal SYBR qPCR Master Mix kit. The reaction conditions on the fluorescence quantitative PCR instrument were set as follows: pre-denaturation at 95 °C for 30 s; 40 cycles at 95 °C for 10 s, followed by 60 °C for 30 s; and a melting curve analysis at 95 °C for 15 s, 60 °C for 30 s, and 95 °C for 15 s. The ΔΔCt method was used to calculate the relative expression level of target genes. The P2X1, P2Y6, α1D and HPRT primers were designed by Sangon Biotech (Shanghai) Co., Ltd., as detailed in Table 1.

**Table 1:** Primer sequences for P2X1, P2Y6, α1D, and HPRT.

Primer name	Primer sequences		bP
P2Y6	Forward primer	TGC​TTG​GGT​GTA​TGT​GGA​GGT	498
	Reverse primer	TGT​TGT​GAA​GTA​GAA​GAG​GAT​A	
P2X1	Forward primer	GAA​GTG​TGA​TCT​GGA​CTG​GCA​CGT	452
	Reverse primer	GCG​TCA​AGT​CCG​GAT​CTC​GAC​TAA	
α1D	Forward primer	CGT​GTG​CTC​CTT​CTA​CCT​ACC	304
	Reverse primer	GCA​CAG​GAC​GAA​GAC​ACC​CAC	
HPRT	Forward primer	CTG​ACC​TGC​TGG​ATT​ACA​TTA	410
	Reverse primer	TTTCGCTGATGACACAA	

### 2.3 Statistical analysis

All statistical analyses and graphic representations were performed using GraphPad Prism 5.0. Experimental data were expressed as the mean ± standard deviation ( $\bar{x}$ ± SD). All data were tested for normality. Differences between multiple groups were compared using one-way analysis of variance (ANOVA) or Kruskal–Wallis H test, while comparisons between two groups were performed using an independent samples *t*-test. A *P* value < 0.05 was considered statistically significant.

## 3 Results

### 3.1 Changes in body weight and systolic pressure in WKY rats and SHR rats before and after exercise training

**Figure 1:**
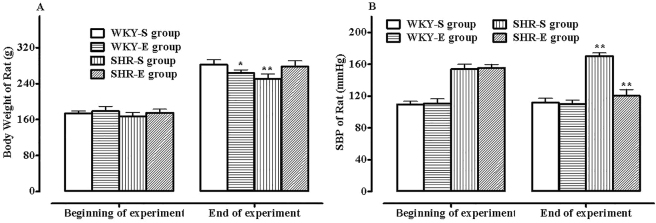
Body weight and changes in systolic pressure in the four groups of rats pre- and post-exercise training. A: Comparison with the WKY-S group (^*^ *P* < 0.05, ^**^ *P* < 0.01); B: Comparison with pre-exercise training values (^**^ *P* < 0.01), *n* = 6.

There was no statistical difference in the pre-exercise training body weight of rats among the four groups (*P* > 0.05). At the end of the experiment, the body weight of rats in all four groups increased to varying degrees, with the SHR-sedentary (SHR-S) group showing the lowest weight gain (*P* < 0.05, Figure 1A). The difference in SBP between the two WKY rat groups before and after exercise training was not significant. However, the effect of exercise on SBP was more significant in SHR rats, with SBP in the SHR-S group increasing significantly over time, while there was a significant decrease in SBP in the SHR-E group compared to pre-exercise training levels, approaching the control group level by the end of the experiment (*P* < 0.01, Figure 1B).

### 3.2 Expression of α1D, P2X1, and P2Y6 receptor mRNA in different arteries of WKY and SHR rats

RT-qPCR was used to quantify the mRNA expression of α1D, P2X1, and P2Y6 receptors in three vascular tissues – RA, Aor, and Cau – in all four groups of rats. The expression level of α1D mRNA in the RA of WKY-S group rats was used as the baseline (expressed as 1.0), with all other expression values presented as relative values.

#### 3.2.1 Expression profiles in WKY rats

**Figure 2:**
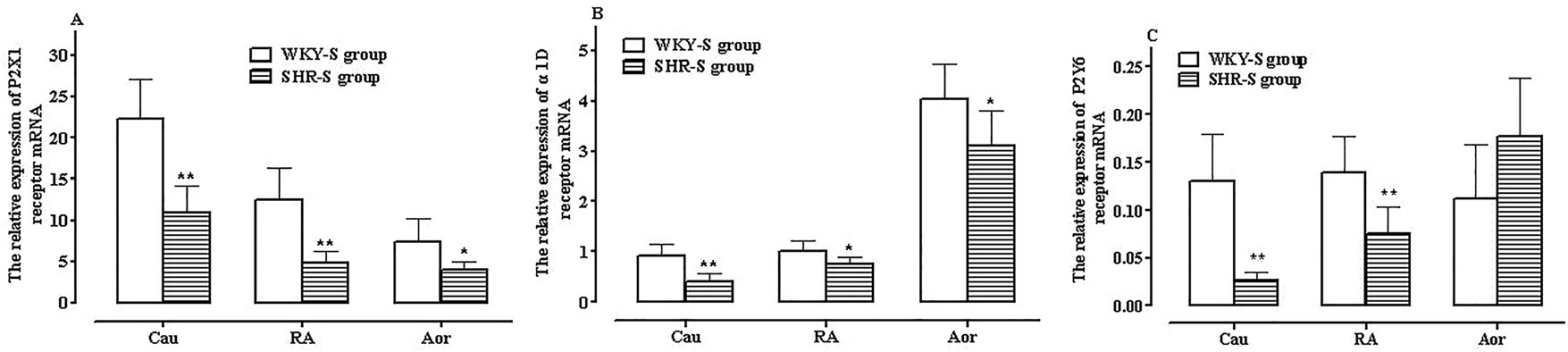
Relative expression of P2X1 (A), α1D (B), and P2Y6 (C) receptor mRNA in RA, Cau, and TA vascular tissues of SHR-S and WKY-S group rats. Comparison with WKY-S group (^*^ *P* < 0.05, ^**^ *P* < 0.01), *n* = 6.

There were significant differences in the expression of α1D, P2X1, and P2Y6 receptor mRNA across the RA, Cau, and Aor in WKY rats. In all three vascular tissues. The relative expression pattern of the three receptor mRNAs was consistent: P2X1 > α1D > P2Y6 (Figure 2A–C).

The expression levels of these receptors also varied among different vascular tissues. P2X1 receptor mRNA expression followed the order: Aor < RA < Cau, with P2X1 expression in the Cau being approximately three times higher than in the Aor (^**^ *P* < 0.01, Figure 2A). The expression of α1D receptor mRNA followed the order: Cau = RA < Aor, with α1D expression in the Aor being approximately four times higher than in the Cau (^**^ *P* < 0.01, Figure 2B). P2Y6 receptor mRNA expression was relatively uniform across the three vascular tissues but significantly lower than P2X1 and α1D expression, (^**^ *P* < 0.01, Figure 2A–C).

#### 3.2.2 Expression profiles in SHR rats

Similar to WKY rats, the expression level of α1D, P2X1, and P2Y6 receptor mRNA in the RA, Cau, and Aor of SHR rats also followed the pattern P2X1 > α1D > P2Y6 (Figure 2A–C).

In different vascular tissues, the expression level of P2X1 receptor mRNA followed the same trend as in WKY rats: Aor < RA < Cau (^**^ *P* < 0.01, Figure 2A); while the expression level of α1D receptor mRNA was highest in the Aor and followed the pattern: Cau = RA < Aor (^**^ *P* < 0.01, Figure 2B). The expression level of P2Y6 receptor mRNA was significantly lower than that of P2X1 and α1D receptors in all three vascular tissues (^**^ *P* < 0.01, Figure 2A–C).

When comparing SHR and WKY rats, P2X1 and α1D receptor mRNA expression levels were reduced to varying degrees in the Cau, RA, and Aor tissues of SHR rats (^*^ *P* < 0.05, ^**^ *P* < 0.01, Figure 2A and B). P2Y6 receptor mRNA expression was significantly decreased in the Cau and RA tissues of SHR rats, whereas it was relatively increased in Aor tissue though the difference was not statistically significant (^**^ *P* < 0.01, Figure 2C).

### 3.3 Effects of long-term exercise on P2X1, α1D, and P2Y6 receptor mRNA expression in WKY and SHR rats

#### 3.3.1 Effects of exercise training on receptor mRNA expression in WKY rats

**Figure 3:**
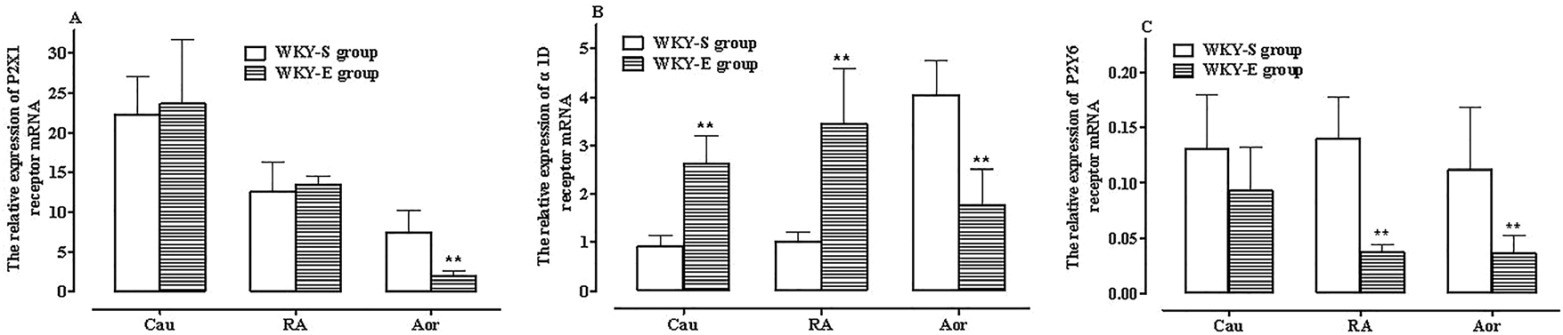
Relative expression of P2X1 (A), α1D (B), and P2Y6 (C) receptor mRNA in RA, TA, and Cau tissues of WKY rats pre- and post-exercise training. Comparison with the control group (^*^ *P* < 0.05, ^**^ *P* < 0.01), *n* = 6.

In the exercise group of WKY rats, the expression levels of α1D, P2X1, and P2Y6 receptor mRNA in the RA and Cau tissues remained similar to pre-exercise training levels, maintaining the relative pattern P2X1 > α1D > P2Y6 (Figure 3A–C). However, in Aor tissue, the expression of α1D and P2X1 receptor mRNA was comparable, deviating from the pre-exercise pattern (Figure 3A and B).

Across the three vascular tissues, the expression level order of P2X1 receptor mRNA followed the order: Aor < RA < Cau, with P2X1 expression in the Cau being approximately 12.5 times higher than in the Aor (^**^ *P* < 0.01). The expression level of α1D receptor mRNA followed the order: Cau = RA > Aor (^**^ *P* < 0.01). Compared to P2X1 and α1D receptor mRNA, P2Y6 receptor mRNA expression remained significantly lower than P2X1 and α1D expression in all three vascular tissues (^**^ *P* < 0.01, Figure 3A–C).

When compared with the sedentary control group WKY rats, exercise exerted different effects on receptor mRNA expression across different vascular tissues: Post-exercise training, P2X1 receptor mRNA expression remained unchanged in the Cau and RA but decreased significantly in the Aor (*P* < 0.01, Figure 3A). In contrast, α1D receptor mRNA expression showed significant differences in all three vascular tissues, with marked increases in the RA and Cau (*P < *0.01) but a significant decrease in the Aor (*P* < 0.01, Figure 3B). P2Y6 receptor mRNA expression decreased in all three vascular tissues, with a significant decrease in RA and Aor tissues (*P* < 0.01, Figure 3C).

#### 3.3.2 Effects of exercise training on receptor mRNA expression in SHR rats

**Figure 4:**
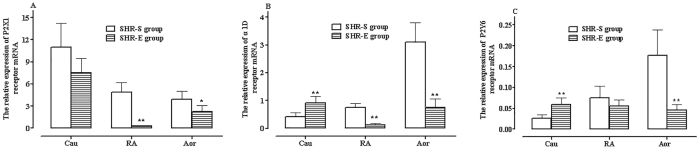
Relative expression of P2X1 (A), α1D (B), and P2Y6 (C) receptor mRNA in RA, TA, and Cau tissues of SHR rats pre- and post-exercise training. Comparison with the control group (^*^ *P* < 0.05, ^**^ *P* < 0.01), *n* = 6.

In the exercise group of SHR rats, the expression levels of P2X1, α1D, and P2Y6 receptor mRNA in the Cau, RA, and Aor tissues was similar to the pre-exercise training levels, all following the pattern P2X1 > α1D > P2Y6 (^**^ *P* < 0.01, Figure 4A–C).

However, the expression of each receptor across different vascular tissues showed distinct changes compared to that before exercise training. The expression level order of P2X1 receptor mRNA changed to Cau > Aor > RA (Figure 4A). There was no significant difference between Cau and Aor α1D receptor mRNA expression, with the lowest expression in RA tissue (^**^ *P* < 0.01, Figure 4B). The expression level of P2Y6 receptor mRNA showed smaller variations across the three vascular tissues and remained significantly lower than that of P2X1 and α1D receptor mRNA (^**^ *P* < 0.01, Figure 4C).

When compared with the sedentary SHR control group, exercise had varying effects on receptor mRNA expression across different vascular tissues: Post-exercise training, P2X1 receptor mRNA expression decreased in the Cau, RA, and Aor of SHR rats, with significant reductions in the RA and Aor (^*^ *P* < 0.05, ^**^ *P* < 0.01; Figure 4A). There was a significant decrease in α1D receptor mRNA expression in the RA and Aor, as well as a significant increase in the Cau (^**^ *P* < 0.01, Figure 4B). P2Y6 receptor mRNA expression increased significantly in the Cau, decreased significantly in the Aor, and showed no significant change in the RA (^**^ *P* < 0.01, Figure 4C).

## 4 Discussion

### 4.1 Distribution of vasoconstrictor receptor mRNA in different arteries of WKY and SHR rats

In the present study, it was found that P2X1 receptor expression was consistently higher than α1D receptor expression in three arteries of WKY and SHR rats. Additionally, P2X1 receptor distribution increased as vascular diameter decreased – a finding consistent with previous research results [9]. Conversely, the α1D transcript is richest in the large-calibre aorta and poorest in the slender caudal artery, implying a positive scaling with luminal diameter. This gradient is not merely numerical: BMY 7378, an α1D-selective antagonist, abolishes the trophic response of aortic smooth muscle to norepinephrine, indicating that the receptor couples chronic sympathetic drive to medial hypertrophy. Consistently, global deletion of the α1D gene reduces wall thickness in both conduit vessels and left ventricle and lowers baseline blood pressure, whereas acute agonist exposure in intact mice evokes a prolonged pressor plateau that outlives plasma catecholamine clearance. Collectively, these data argue that α1D receptors act as diameter-dependent mechanochemical amplifiers, translating transient sympathetic bursts into sustained myogenic tone and structural reinforcement, thereby stabilizing perfusion pressure across organs perfused by vessels of unequal caliber [10].

Compared to WKY rats, SHR rats showed reduced expression of both P2X1 and α1D receptors in Cau, RA, and Aor tissues. Given that downregulation of P2Y1 and P2Y2 receptors in these vascular tissues was identified in earlier studies [9], it is plausible that SHR rats experience pathological alterations in both vasoconstrictive and vasodilatory receptor distribution, potentially contributing to hypertension-associated vascular dysfunction. Taken together, it can be inferred from the results of this study that this differential and dominant distribution of P2X1 receptors may be beneficial for the body in terms of regulating peripheral vascular tone, improving vascular contractility, and facilitating blood redistribution in different organs under pathological conditions such as shock or hypovolemia. This mechanism likely serves to preserve vital organ function. Meanwhile, the distribution characteristics of α1D receptors in various vascular tissues appear essential for maintaining vascular tone and circulatory stability.

### 4.2 Effects of exercise training on vasoconstrictor receptor mRNA in WKY and SHR rats

It has been demonstrated that regular aerobic exercise effectively lowers both dynamic blood pressure and resting systolic pressure, particularly in cases of resistant hypertension. In the present study, the effect of exercise was also observed in SHR rats, there was a significant decrease in SBP in the SHR-E group. And it was seen that long-term exercise training preserved the distribution characteristics of contractile vascular receptors in different vascular tissues of both SHR and WKY rats, particularly maintaining the dominant distribution of P2X1 receptors. However, exercise was found to exert a more significant impact on adrenergic α1D receptors than on purinergic P2X1 receptors across vascular tissues in both rat strains. Specifically in WKY rats, exercise led to the most significant increase in α1D receptor expression in Cau and RA tissues of WKY rats, while its effect on P2X1 receptors was not significant.

It was also noted that exercise-induced changes were more pronounced in SHR rats than in WKY rats. In SHR rats, exercise resulted in the increased expression of α1D and P2Y6 receptor mRNA in the Cau, while all other receptor mRNAs in the remaining vascular tissues showed decreased expression. It has been reported that during moderate to high-intensity exercise and at rest, the sympathetic vasoconstrictive effects mediated by *α*-adrenergic receptors in inactive skeletal muscle vessels are dominant and crucial for maintaining blood pressure. However, the metabolic activities during exercise can modulate the function of *α*-adrenergic receptors. Both sympathetic vasoconstriction and nitric oxide (NO)-mediated sympatholytic vasodilation during exercise require the participation of α1-adrenergic receptors.

The observed exercise-induced changes in vascular receptor expression may be associated with modifications in contractile function of local blood vessels during exercise, potentially contributing to enhanced cardiovascular regulation and optimized blood supply to essential organs during physical exertion.

### 4.3 Effects of exercise training on P2Y6 receptor distribution in different vascular tissues of WKY and SHR rats

The constitutively low P2Y6 transcript detected in caudal, renal and aortic segments of both strains places this UDP/UTP-sensing receptor outside the dominant vasoconstrictor axis defined by P2X1 and α1D. Yet its divergent, vessel-specific response to exercise – up-regulation in the SHR caudal artery while down-regulated in conduit vessels – suggests a context-dependent switch from a minor contractile role to a modulator of local trophic signalling. This interpretation aligns with observations in P2Y6^−/−^ mice that exhibit markedly attenuated atherosclerosis and reduced lipid retention in the abdominal aorta; absence of the receptor appears to blunt pro-inflammatory cytokine release and macrophage recruitment without compromising baseline tone. Collectively, the data imply that exercise-induced increases in P2Y6 within small resistance arteries of hypertensive animals may transiently facilitate nucleotide-mediated vasoconstriction or remodel the extracellular matrix, whereas down-regulation in larger vessels could limit maladaptive inflammatory cascades that precede atherogenesis.

Currently, there are no other reports on the effects of exercise training on vascular P2Y6 receptors. Therefore whether the low baseline expression of P2Y6 receptors in these three arterial vessels or their exercise-induced changes contribute to alterations in vasoconstriction and vasodilation function requires further investigation. Moreover, while our preliminary analysis suggested a potential link between these two variables, the significance was not robust, likely due to the limited sample size or the influence of other confounding factors. Despite the lack of a strong statistical correlation, we believe these findings still provide valuable insights and lay the groundwork for future investigations with larger sample sizes and more sophisticated analyses.

In summary, compared to α1D receptors, similar dominant distribution of P2X1 receptors distribution were demonstrated across all examined blood vessels in WKY rats and SHR rats. The distribution of these receptors was closely associated with vascular diameter, with P2X1 receptors predominantly expressed in the Cau – the smallest-diameter artery – suggesting a high abundance of functional P2X1 receptors on its vascular smooth muscle. In contrast, α1D adrenergic receptors were most abundant in the Aor, a large-diameter artery. SHR rats showed significantly lower expression of P2X1 receptors, α1D receptors, and P2Y6 receptor mRNA across all arterial tissues compared to WKY rats. Given that decreased expression of P2Y1 and P2Y2 receptors in rat vasculature has been reported in previous research, it can be inferred from these findings that both vasoconstrictive and vasodilatory receptors may undergo pathological changes in SHR rats.

Post-exercise training, P2X1 receptor mRNA expression remained dominant in all arteries of both rat strains; however, exercise was found to exert a more pronounced effect on α1D receptor expression than P2X1 receptor expression, significantly increasing its mRNA levels in Cau and RA tissues of WKY rats, as well as statistically increased mRNA expression of α1D receptor in the Cau of SHR rats. On the contrary, the expression of all other receptors declined in varying degrees in the remaining vascular tissues of WKY and SHR rats. Based on these results, combined with previous experimental findings on vascular P2Y receptors, it can be inferred that exercise-induced changes receptor distribution may be related to local contractile function during exercise. These adaptations could play a role in cardiovascular function regulation and optimal blood supply to vital organs during physiological stress induced by exercise.
